# Short-term labour transitions and informality during the COVID-19 pandemic in Latin America

**DOI:** 10.1186/s12651-023-00342-x

**Published:** 2023-05-17

**Authors:** Roxana Maurizio, Ana Paula Monsalvo, María Sol Catania, Silvana Martinez

**Affiliations:** 1 Instituto Interdisciplinario de Economía Política, Universidad de Buenos Aires-CONICET, Buenos Aires, Argentina; 2 Universidad Nacional de General Sarmiento, Los Polvorines, Argentina; 3 Instituto Interdisciplinario de Economía Política, Universidad de Buenos Aires, Buenos Aires, Argentina

**Keywords:** COVID-19, Latin America, Labour transitions, Informality, J01, J08, N36

## Abstract

Latin America was one of the regions hardest hit by the COVID-19 pandemic. This paper analyses, from a dynamic and comparative perspective, labour transitions triggered by the pandemic in six Latin American countries: Argentina, Brazil, Costa Rica, Mexico, Paraguay, and Peru. Special attention is paid to transits around labour informality during this period. Unlike previous crises, the fall in informal occupations deepened the overall contraction in employment. This was explained by a significant increase in exit rates from these jobs and, to a lesser extent, by reductions in entry rates. Most of the informal workers who lost their jobs left the labour force. Contrary to this labour movement, transits from informal to formal jobs significantly dropped during the most critical phase in this crisis. Partial recovery in employment since mid-2020 has been led by an increase in informal jobs. The labour dynamic has been different between men and women. This study reveals the relevance of dynamic analysis to clearly identify labour transitions that occurred during a labour crisis of unprecedented intensity and characteristics in Latin America.

**Supplementary Information:**

The online version contains supplementary material available at 10.1186/s12651-023-00342-x.

## Introduction

The COVID-19 pandemic has caused an unprecedented economic recession in Latin America given its scale and spread. In 2020, Latin American GDP shrank by 6.8%, representing the biggest regional economic crisis ever recorded since the early twentieth century (ECLAC 2021a). The pandemic broke out in a region already characterised by an economic slowdown or reversal of labour improvements achieved over the previous years, where labour informality and inequality were still high, and social welfare and health systems, very weak. Given this context, the crisis struck harder in employment than in economic activity and the impact was highly unequalizing because informal workers, low-skilled workers and women were disproportionately affected by it.

This article analyses, from a dynamic and comparative perspective, short-term labour transitions triggered by the pandemic in six Latin American countries: Argentina, Brazil, Costa Rica, Mexico, Paraguay, and Peru. Special attention is paid to transits around labour informality. In particular, we analyse the transition matrices between different labour statuses, the entry and exit rates from and into formality and informality, and the probabilities of transition for different groups of people. The labour dynamics experienced by women and men are evaluated. To the best of our knowledge, this study represents the first attempt to evaluate the labour market effects of the pandemic in a wide set of Latin American countries from a dynamic perspective, particularly focusing on labour informality.

Three aspects of the paper are worth highlighting. First, this study uses information on labour transitions besides the most traditional cross-sectional data. This dynamic approach contributes to deepening the knowledge of the impacts of the pandemic on Latin American labour markets, since it allows us to identify the intensity and direction of the labour flows during this crisis. This aspect becomes even more relevant given the high occupational turnover that characterises Latin America (Beccaria and Maurizio 2020).

Second, this article deals with a relevant topic because informality is one of the most distinctive features of labour markets in this region. In the fourth quarter of 2019, almost 50% of workers held informal occupations (ILO 2021b). The informality rate was even higher in three out of the six countries under study: Mexico (55%), Paraguay (69%) and Peru (71%). Argentina (45%), Brazil (39%) and Costa Rica (43%) stood below the regional average, though these rates were also high. Informality, in turn, is an important source of occupational instability in Latin America (Beccaria and Maurizio 2020). Moreover, in this crisis, informality behaved differently when compared to previous crises because it did not play its typical counter-cyclical role. Therefore, the analysis of flows from and into labour informality enables us to characterise in greater detail the dynamic of this type of occupation throughout the period under analysis.

Third, a comparative analysis is performed involving six Latin American countries. The selection of this set of countries provides a broad picture of the labour impacts of COVID-19 in Latin America, as it makes possible the consideration of cases with different occupational and income structures. At the same time, since the largest countries are included, this selection accounts for about 70% of the overall population in the region. However, given the high heterogeneity between these labour markets, the descriptive and econometric results are presented separately for each country and then a comparative analysis among them is carried out.

This paper uses microdata from regular household surveys conducted by each country’s national institutes of statistics. Although these surveys are not longitudinal, their rotating panel schemes allow short-term flow data to be drawn from them. In addition to the descriptive analysis of transitions matrices, econometric regressions were run to estimate the probabilities of transitions between formal and informal occupations, unemployment and inactivity.

Our results show that, in contrast to previous crises, the drop in informal occupations magnified the overall employment contraction. This was driven by a notable rise in exit rates from this job type, and to a lesser degree, by a drop in entry rates. In most of the countries studied, stability gaps between formal and informal occupations broadened. Most of the informal workers who lost their jobs, left the labour force. On the other side, transits from informal to formal jobs reduced significantly during the most critical point of this crisis. The evident greater job losses suffered by women during this period were not explained by a more intense increase in the probabilities of losing an informal job in comparison with men. Rather, the more precarious employment composition and increasing constraints on women's labour supply explain this result. Symmetrically, during the subsequent recovery phase—since mid-2020—the rise in total employment was led by a rise in informal jobs. The drop in the intensity of exit rates from these occupations mostly explained this behaviour, although a greater intensity in entry flows to informality from inactivity also accounted for this trend.

The paper is organised as follows. Section 2 presents the literature review. Section 3 describes the sources of information and methodologies used. Section 4 details the definition and measurement of labour informality. Section 5 presents an overview of the labour crisis triggered by COVID-19 in the six countries under study. Section 6 analyses the labour exit and entry rates and labour destinations after leaving an informal job. Section 7 presents the assessment of attrition and its correction. Finally, Sect. 8 concludes.

## Literature review

This section reviews the empirical evidence for Latin America in relation to three strands of literature closely linked to the main issues addressed in this paper: (1) the labour formalization process before the pandemic; (2) labour market transitions before the pandemic and (3) labour market performance and labour transitions during the pandemic.

### Labour formalization process in Latin America before the pandemic

Salazar-Xirinachs and Chacaltana (2018) argue that one of the most significant changes in Latin American labour markets since the turn of the century has been the process of formalization. During the decade of 2005–2015, the region created 51 million jobs, of which 39 million were formal jobs, leading to a reduction in the informal employment rate over this period. In Argentina, formal jobs rose by almost 60% between 2003 and 2017, while total employment increased by 20%. In Brazil, these figures were 40% and 20% respectively. In Ecuador, Paraguay and Peru the number of formal jobs more than doubled during this period (Maurizio and Vázquez 2019). An exceptional expansionary economic cycle, along with a range of policy interventions—such as labour inspection, incentives, simplified registration, and tax reductions—resulted in this process of formalization (Berg 2010; Bertranou and Casanova 2013; Maurizio 2015; Salazar-Xirinachs and Chacaltana 2018).

Several studies have examined the characteristics of this regionally observed phenomenon. Bertranou et al. (2013) and Maurizio (2015) found that formalization was more intense among salaried men, working full-time and in big companies. Maurizio (2015) found that informal workers who were initially located in the upper part of the wage distribution had the highest probabilities of becoming formal. This result is consistent with the fact that formalization was more intense among workers with a “better” vector of observable attributes, such as a high educational level or job tenure. Maurizio and Vázquez (2019) confirmed all these findings in five Latin American countries: Argentina, Brazil, Ecuador, Paraguay and Peru.

Other studies have analysed the distributive impact of the formalization process (Amarante and Arim 2015; Beccaria et al. 2020; Maurizio 2015; Beccaria et al. 2015). One common result is the positive correlation between the reduction in informality and the fall in wage inequality. ECLAC and ILO (2014) studied the impact of this formalization process on gender wage gaps, obtaining mixed results. In Brazil, Ecuador, Panama and Paraguay the increase in formality narrowed the wage gap between men and women, while the opposite result appears in Bolivia and Colombia. As Maurizio et al. (2021) showed, however, the formalization process has slowed down or even reversed since 2014/2015. The formality rate declined between these years and 2019 in many countries of the region (− 2 pp in Argentina, − 4 pp in Ecuador, − 3.5 pp in Brazil) or remained constant in others (Peru and Paraguay). On average, in 2019 the informality rate in Latin America was 50%, similar to that in 2011/2012 (ILO, 2021b).

### Labour market transitions before the pandemic

In addition to informality, the high occupational turnover is another structural characteristic of Latin American labour markets. Donovan et al. (2020) found that job-finding rates, employment exit rates and job-to-job transition rates are higher in low/middle-income countries (including Argentina, Bolivia, Brazil, Costa Rica, Ecuador, Mexico, Nicaragua, Paraguay, and Peru) than in high-income countries. In this line, Beccaria and Maurizio (2020) concluded that labour turnover rates in Latin American countries (Argentina, Brazil, Ecuador, Mexico, Paraguay, and Peru) are greater than those seen in Great Britain, Germany and the United States. Higher exit rates from jobs in these Latin American countries are only partially associated with larger incidences of informal and fixed-term contracts, since formal jobs in these countries are also more unstable than in developed countries.

Castillo et al. (2006) found that the average retention rate of formal salaried workers in Argentina between 1996 and 2004 was very low, with approximately half of all formal workers in 1996 no longer employed in formal jobs in 2004. The situation was worse for informal workers, who faced an even higher occupational turnover. Maurizio et al. (2021) concluded that informal workers are not only less likely to remain in an occupation, but also are more likely to move to another informal job after leaving their initial occupation. In the same vein, Beccaria and Maurizio (2020) found that while 60% to 80% of formal workers who exit their job go on to another formal job or become professional own-accounts or employers, this percentage is significantly lower (20%/40%) among informal workers.

### Latin American labour market performance and labour transitions during the COVID-19 pandemic

The dramatic fall in the level of economic activity in Latin America during 2020—about 7%—impacted employment with an unprecedented intensity. The employment rate fell by 10%, resulting in an extremely high employment-product elasticity,^1^ almost 1.5. The pandemic, the lockdown and social-distancing measures led to a series of unprecedented labour responses that accounted for such behaviour. In particular, during the contraction phase labour informality did not play the traditional counter-cyclical role, but rather intensified the overall fall in employment by dropping more intensely than formal occupations. Therefore, unexpectedly, the regional informality rate contracted sharply in the midst of the crisis. Workers who lost their jobs did not remain in the labour force, as is usual in the region, but became inactive (Beccaria et al. 2022; ILO, 2020b, 2021a; Maurizio 2021; Velasco 2021; ECLAC and ILO, 2020; Velásquez 2021; Acevedo et al. 2021).

In addition to affecting informal workers, the COVID-19 crisis has also had a more severe impact on women than men (ILO 2020a, b, 2021a; Berniell and de La Mata 2021; Bergallo et al. 2021). At least three factors are associated to this result. First, women faced greater difficulties in reconciling paid work with care activities in the face of the closure of schools and childcare services (Velasco 2021; ECLAC 2020a, b); second, the measures implemented during lockdowns particularly affected female sectors of activity, such as hotels and restaurants; third, women suffer in general from a higher level of informality compared to men (ILO 2020a).

From the second half of 2020, economic recovery allowed employment to reverse the downward trend. This growth was strongly driven by informal jobs (ILO 2020a, b, 2021a) which implied that the regional informality rate had returned to the values of 2019. Together with informal workers, women also experienced more intense employment recovery (ILO 2021a). By the end of 2021, the employment gender gap was slightly above pre-pandemic levels (ILO 2021b).

Few studies have analysed labour transitions in Latin American countries during the COVID-19 crisis. According to Soares and Berg (2022), during the first two quarters of 2020, employment retention rates were higher for the three European countries considered (Poland, Portugal and Great Britain) than for Brazil, Costa Rica, Mexico and the United States. In all of the seven countries under study, the COVID-19 pandemic deepened labour market inequalities as women, young and low-skilled workers faced the lowest probabilities of keeping their jobs. At the opposite end, full-time, permanent, and formal workers had the lowest risk of losing their jobs. For Peru, Chacaltana et al. (2021) showed that during the COVID-19 pandemic the labour market was very volatile, and most transitions were between employment and inactivity; in particular, from informal employment to inactivity during the first half of 2020 and from inactivity to informality for the remainder of the year. Again, women, young, elderly, and low-skilled workers held the lowest chances to be employed. In addition, the likelihood for informal workers to transit to a formal job decreased by about a third.

Building on previous studies, this paper contributes to fill the knowledge gap on the intensity and characteristics of the labour market transitions that occurred during the COVID-19 crisis in Latin American countries. Specifically, our analysis, which is updated to the third quarter of 2021, enables us to compare the labour flows observed during the contraction and recovery phases. Furthermore, the inclusion of a large number of countries in our study provides in-depth evidence of common patterns and heterogeneity across the region. By focusing on informality and gender, this paper sheds light on the labour dynamics of the two groups of workers most affected by the pandemic. Finally, our econometric estimates, combined with attrition correction, yield robust results and valid conclusions.

## Data and methodology

### Data

The analysis covers the period between the first quarter of 2019 and the third quarter of 2021. This timeframe enables us to obtain information prior to the outbreak of the pandemic and almost two years after its onset. Data used in this paper come from regular household surveys conducted by each country’s national institutes of statistics.

In Argentina, the Encuesta Permanente de Hogares (EPH) is carried out quarterly in 31 urban areas by the National Institute of Statistics and Census (INDEC). In Brazil, the Brazilian Institute of Geography and Statistics (IBGE) conducts the Pesquisa Nacional por Amostra de Domicílios *Contínua* (PNADC), and nationally representative data are released every three months. In Costa Rica, the Encuesta Continua de Empleo (ECE) is conducted quarterly by the National Institute of Statistics and Census (INEC) and it has national coverage. The source of information used for Mexico is the Encuesta Nacional de Ocupación y Empleo (ENOE). This survey is carried out by the National Institute of Statistics and Geography (INEGI) and it is nationally representative. There are ENOE surveys available for all of 2019 and for the first, third and fourth quarters of 2020. During the second quarter of 2020, the Encuesta Telefónica de Ocupación y Empleo (ETOE) was conducted over the phone. The ETOE’s sample is smaller, about 10% of ENOE’s. However, there are enough observations to reconstruct the labour trajectories over the period under analysis. In Paraguay the Encuesta Permanente de Hogares Continua (EPHC) is carried out quarterly by the National Office of Statistics, Surveys and Census (INE). It has national coverage. Finally, in the case of Peru, the source of information is the Encuesta Permanente de Hogares (EPE) conducted by the National Institute of Statistics and Computing (INEI). Its geographical coverage includes Metropolitan Lima and Callao.

Although these surveys are not longitudinal, their rotating panel sample design allows flow data to be drawn from them. In such a scheme, the sample is composed of a specific number of groups of households (rotation groups), which are brought in and out from the sample according to a specific pattern. Consequently, it is possible to trace changes for a given proportion of the sample along the period under analysis. Table 1 details the rotational panel design in each of these six countries. Considering these schemes, panels were built with data from two consecutive quarters for Argentina, Brazil, Costa Rica and Mexico; and from year-over-year data for Paraguay and Peru. Due to different observation windows, the intensity of labour flows between the first and second group of countries cannot be directly compared. Nevertheless, we can still evaluate how the outbreak of the pandemic impacted the magnitude and direction of labour movements in each country, as compared to the pre-pandemic period.

**Table 1:** Rotation panel schemes

Country	Survey	Rotation panel schemes		TOR (*)
Argentina	EPH	Q1 Q2 (Q3 Q4) Q5 Q6	The individual is interviewed for two consecutive quarters, excluded from the survey for two quarters, and interviewed two more times	50% (a)
Brazil	PNADC	Q1 Q2 Q3 Q4 Q5	The individual is interviewed for five consecutive quarters	80% (a)
Costa Rica	ECE	Q1 Q2 Q3 Q4	The individual is interviewed for four consecutive quarters	75% (a)
Mexico	ENOE–ETOE	Q1 Q2 Q3 Q4 Q5	The individual is interviewed for five consecutive quarters	80% (a)
Paraguay	EPHC	Q1 (Q2 Q3 Q4) Q5	The individual is interviewed in one quarter, exits the survey for three consecutive quarters, and returns in the fifth quarter for a final interview	50% (b)
Peru	EPE	Q1 (Q2 Q3 Q4) Q5	The individual is interviewed in one quarter, exits the survey for three consecutive quarters, and returns in the fifth quarter for a final interview	50% (b)

(a) Panel data from two consecutive quarters, (b) Panel data from the same quarter in two consecutive years. *Theoretical overlap rate

Source: Own elaboration based on official information from national institutes of statistics

The final sample is composed by individuals of over 15 years of age, thus taking into consideration the lower age limit as set by the International Labour Organisation (ILO) to define the working-age population. Individuals with missing data were removed from the sample. Table 2 presents, for each country and panel, the number of cross-sectional data observations in the first sample, the number of matched observations (panel data), and the proportion of these observations over initial cross-sectional data (proportion of actual overlap).

**Table 2:** Number of observations in cross-sectional and panel data

Q(t)	Argentina			Brazil		
	Cross-sectional data Q(t)	Panel data Q(t)-Q(t + 1)	% actual overlap	Cross-sectional data Q(t)	Panel data Q(t)-Q(t + 1)	% actual overlap
I19	59,369	21,881	37%	553,308	380,673	69%
I20	51,643	15,038	29%	487,937	288,786	59%
II21	46,996	15,988	34%	356,239	246,355	69%

Q(t)	Costa Rica			Mexico		
	Cross-sectional data Q(t)	Panel data Q(t)-Q(t + 1)	% actual overlap	Cross-sectional data Q(t)	Panel data Q(t)-Q(t + 1)	% actual overlap
I19	25,695	15,743	61%	406,036	283,580	70%
I20	24,746	18,684	76%	417,783	9183	2%
II21	23,669	14,585	62%	381,319	202,971	53%

Q(t)	Paraguay			Peru		
	Cross-sectional data Q(t)	Panel data Q(t)-Q(t + 1)	% actual overlap	Cross-sectional data Q(t)	Panel data Q(t)-Q(t + 1)	% actual overlap
I19	11,933	4110	34%	14,550	4879	34%
II19	16,653	12,203	73%	13,462	5764	43%
III20	9753	3416	35%	14,470	4455	31%

“% actual overlap” corresponds to the proportion of matched observations over total initial cross-sectional data

Source: Own elaboration based on household surveys

### Estimation of transition probabilities

The Markovian process is the methodological framework for the study of transitions between labour market statuses. If the process is represented by a first order model, then the probability to change from status *i* to status* j* (*p*_*ij*_) is homogeneous among individuals and constant in time.^2^ In other words, transition probability is equal for all those in status *i* at a time *t*, regardless of their status in prior periods. Under these conditions, transition probabilities are consistently estimated from the observed transitions*.* Assuming labour status groups represent a partitioned space into finite mutually-exclusive events, the probability to move from state *i* in time *t* to state *j* in time *t* + *1* (exit probabilities) is^3^:

1 $${p}_{ij}=\frac{{N}_{ij}}{{N}_{i}}\quad \mathrm{with }\quad \sum_{j}{p}_{ij}=1$$

where i and j correspond to labour status groups: formal worker, informal worker, unemployed, out of the labour force. *N*_*i*_ is the population that was in status *i* at time *t*, and* N*_*ij*_ is the population that was in status *i* in period *t* and it moves to j in *t* + *1.*

Then, logit regression models were estimated to assess the impacts of labour and socioeconomic characteristics on exit probabilities. We ran these models [Eq. 2] in two cases. First, to evaluate the effect of informality on job exit probability. Second, to assess the impact of gender on exit probabilities from an informal job.

2 $${p}_{ij}=P(Y=1|X)=\frac{{e}^{\beta X}}{1+{e}^{\beta X}}$$

In the first case the dependent variable Y is a binary indicator that takes value 1 when the worker exits employment and 0 when he/she remains working. The covariates are age, level of education, gender, household position, marital status, presence of children in the household, region, branch of activity, size of the firm, informality and time indicators. The model incorporates interaction terms between time indicator and informality. In the second case the dependent variable Y is a binary indicator that takes value 1 when an informal worker leaves their occupation and 0 when he/she remains in informality. The covariates are the same as the first model except that informality and the interaction between informality and period were removed, and that the interaction between gender and period was added.

Finally, multinomial logistic regression models were performed to estimate the probabilities of exit from informal employment ($${p}_{informal, j}$$) to different destinations ($$j)$$[Eq. 3]. The dependent variable Y has K categories, corresponding to each of the possible work status destinations once an informal worker leaves their job (formal employment, unemployment or inactivity). The covariates are age, education, gender, household position, marital status, presence of children in the household, region, branch of activity, size of the firm, and period.

3 $${p}_{informal, j}=P\left(Y=j |X\right)=\frac{{e}^{{\beta }_{j}X}}{1+{\sum }_{k\ne j}{e}^{{\beta }_{k}X}}$$

### Calculation of the contribution of entry and exit flows to variations in employment

The net variation in the proportion of employment in total individuals between the two waves considered in each panel is written as:

4 $$\Delta {E}^{{P}_{i}}=\left({E}_{{w}_{2}}^{{P}_{i}}- {E}_{{w}_{1}}^{{P}_{i}}\right)$$

where $(\Delta {E}^{{P}_{i}})$ is the net variation in the proportion of employment in total individuals between wave 1 and wave 2; ${E}_{{w}_{1}}^{{P}_{i}}$ is the proportion of employed people in the initial wave (w_1_) of the total people included in the panel *i*; ${E}_{{w}_{2}}^{{P}_{i}}$ is the proportion of employed people in the final wave (w_2_) of the total people included in the panel *i*.

In turn, $\Delta {E}^{{P}_{i}}$ is the result of the difference between employment inflows (entries) and employment outflows (exits):

5 $$\Delta {E}^{{P}_{i}}=\left({E}_{{w}_{2}}^{{P}_{i}}- {E}_{{w}_{1}}^{{P}_{i}}\right)={En}_{{w}_{2}{w}_{1}}^{{P}_{i}}-{Ex}_{{w}_{2}{w}_{1}}^{{P}_{i}}$$

where ${En}_{{w}_{2}{w}_{1}}^{{P}_{i}}$ is the proportion of non-employed people entering employment between the two waves of the total people included in the panel; ${Ex}_{{w}_{2}{w}_{1}}^{{P}_{i}}$ is the proportion of employed people exiting employment between the two waves of the total people included in the panel.

For each of the two panels considered we have:

6 $$\begin{aligned}\Delta {E}^{{P}_{1}}=\left({E}_{{w}_{2}}^{{P}_{1}}- {E}_{{w}_{1}}^{{P}_{1}}\right)={En}_{{w}_{2}{w}_{1}}^{{P}_{1}}-{Ex}_{{w}_{2}{w}_{1}}^{{P}_{1}}\\\Delta {E}^{{P}_{2}}=\left({E}_{{w}_{2}}^{{P}_{2}}- {E}_{{w}_{1}}^{{P}_{2}}\right)={En}_{{w}_{2}{w}_{1}}^{{P}_{2}}-{Ex}_{{w}_{2}{w}_{1}}^{{P}_{2}}\end{aligned}$$

After rearranging terms, the following equation is obtained:

7 $$\underbrace{\Delta {{E}^{{{P}_{2}}}}-\Delta {{E}^{{{P}_{1}}}}}_{\Delta \Delta E}=\underbrace{\left( En_{{{w}_{2}}{{w}_{1}}}^{{{P}_{2}}}-En_{{{w}_{2}}{{w}_{1}}}^{{{P}_{1}}} \right)}_{\Delta En}-\underbrace{\left( Ex_{{{w}_{2}}{{w}_{1}}}^{{{P}_{2}}}-Ex_{{{w}_{2}}{{w}_{1}}}^{{{P}_{1}}} \right)}_{\Delta Ex}$$

8 $$1=\frac{\Delta En}{\Delta \Delta E}-\frac{\Delta Ex}{\Delta \Delta E}$$

The last equation computes to what extent changes in the intensity of entry flows and exit flows contributed to the evolution of total employment during the period under analysis.

### Attrition bias identification and correction

A potential limitation of panel data is that the proportion of individuals actually interviewed in two successive periods (actual overlaps) may be less than expected according to the theoretical sample rotation scheme. This loss of observations (attrition) can generate a sample bias if it is not random. Therefore, an assessment of the presence of non-random attrition and its correction are needed. The methodology to do so comprises three consecutive stages: identification of attrition, evaluation of random/non-random nature of attrition, and correction of the sample bias if attrition is non-random and affects the variable under study. Implementing these steps requires having a variable in the survey that identifies if the individual drops out of the survey earlier than would correspond according to the theoretical rotation scheme. This evidences the presence of attrition. Due to a lack of this variable in the household surveys used in Argentina, Costa Rica and Peru, this procedure was only possible for Brazil, Mexico and Paraguay. The appendix presents a detailed description of these three stages.

## Definition and measurement of labour informality

Labour informality is the key dimension studied in this paper. The definition of informal employment is based on the recommendations from the Conferences of Labour Statisticians (CIETs). Accordingly, informal salaried workers are those whose jobs are not subject to national labour laws. Non-salaried workers are considered informal if they carry out their activities in the informal sector.

In order to implement this approach to informality in the six countries considered, the criteria of the ILO’s Regional Office for Latin America and the Caribbean were followed to obtain comparable data, taking into account the availability of information in each of the surveys used (Additional file 3: Table S1). In some countries, informal wage earners are those whose employers do not contribute to the social security system (pensions and/or health) on their behalf. In others, informal wage earners are those without a labour contract. Absences of enterprise registration with certain public institutions or tax agencies, as well as the nonexistence of bookkeeping are used to identify the informal sector. If this information is not reported, other proxies, such as the place where the activities are carried out (e. g., in the streets) and/or the size of the establishment are used.

## Overview of the labour crisis triggered by COVID-19 in the countries under analysis

### Evolution of the main labour market indicators

From the onset of the pandemic up to the third quarter of 2021, it is possible to identify two contrasting phases in the Latin American labour market and, in particular, in the countries under analysis (Table 3). During the first phase (IVQ2019-IIQ2020) the employment rate strongly dropped in all six countries: Paraguay (− 10%), Brazil (− 12%), Mexico (− 20%), Argentina (− 20%), Costa Rica (− 21%), and Peru (− 41%). This was the result of a drop in economic activity during this period, ranging from − 8% in Paraguay to − 31% in Peru-, coupled with specific characteristics of these labour markets, which will be explored further later.

**Table 3:** Evolution of main labour market indicators

	Argentina			Brazil			Costa Rica		
	IV19	II20	III21	IV19	II20	III21	IV19	II20	III21
Employment rate	55	44	56	57	50	55	55	44	52
Unemployment rate	9	13	8	11	14	13	12	14	15
Participation rate	60	50	61	65	58	63	63	58	61
Informality rate	45	34	44	39	35	39	43	37	41
Economic activity index	100	81	102	100	88	100	100	90	104

	Mexico			Paraguay			Peru		
	IV19	II20	III21	IV19	II20	III21	IV19	II20	III21
Employment rate	57	46	57	69	62	67	72	42	68
Unemployment rate	3	5	4	6	8	7	5	17	7
Participation rate	59	48	59	73	67	71	75	51	73
Informality rate	54	50	54	64	61	63	71	72	73
Economic activity index	100	81	97	100	92	102	100	69	102

Economic activity index IV2019 = 100

Source: Own elaboration based on household surveys and statistical institutes

In all of these cases, the fall in employment led to transits into unemployment, but mostly strong outflows from the labour force (Table 3). The reduction in the participation rate went from − 8% (Costa Rica) to − 33% (Peru). This was due to both unfavourable expectations regarding the likelihood of finding a job, as well as the lockdown and social-distancing measures that limited opportunities for job searching. In fact, there is a close positive correlation between the Oxford Stringency Index^4^ (Hale et al. 2021) and the intensity of labour force reduction. Argentina and Peru—with a sharp reduction in labour participation- were the first two of the six countries under analysis to implement Covid regulation, and did so with great intensity. Contrastingly, Brazil and Costa Rica imposed less restrictive measures and the labour supply fell less. These exit flows out of the labour force strongly moderated the impact of the loss of employment on the unemployment rate, which, in any case, increased in all six countries. In Peru, for instance, this rate rose from 5 to 17%.

By mid-2020, Latin America -and in particular the countries under analysis- began a path of economic and labour market recovery on the back of good vaccination rates and control of the health situation. As restrictions gradually eased, people who were previously out of the labour force began to work, and others actively started job searching. Consequently, the employment rate and economic participation rate both exhibited an upward trend, while unemployment rates declined (Table 3).

However, the recovery phase was not intense enough to return to pre-pandemic values. Only Argentina saw a higher employment rate in the third quarter of 2021 than that in the fourth quarter of 2019, while in Mexico this indicator was the same as before the pandemic. At the opposite end, in Peru and Costa Rica, the employment rate was between 5 and 6% lower than 2019. In Brazil and Paraguay, the employment gap was somewhat smaller, − 4.3% and − 2.9%, respectively (Table 3). In conclusion, nearly two years into the pandemic, and with the exception of Argentina and Mexico, the recovery of employment was still incomplete as of the third quarter of 2021.

### Evolution of labour informality

As mentioned before, both formal and informal employment experienced significant contractions during the crisis, with the latter being more severely impacted (ILO 2020b; Beccaria et al 2022). Thus this “traditional adjustment mechanism” greatly weakened. This is observed in all the countries considered in this paper, apart from Peru, where formal occupations fell more than the informal ones. In the other five countries, the highest contraction in informal employment resulted in a (temporary) decline in the informality rate, with a decrease of 11 pp in Argentina, 7 pp in Costa Rica, 5 pp in Mexico, 4 pp in Brazil and 3 pp in Paraguay (Additional file 1: Fig. S1). Consequently, the decrease in informal occupations during the first half of 2020 accounted for between 55% (in Brazil) and 85% (in Argentina and Paraguay) of the overall employment reduction (Additional file 2: Fig. S2). The factors which can explain the higher contraction in informal occupations compared to formal ones are discussed later.

As from the second half of 2020, the partial recovery in employment was driven by a rise in informal occupations (Additional file 1: Fig. S1), explaining from 58 to 78% of net job creation (Additional file 2: Fig. S2). This implied, in all cases, an increase in the informality rate during this subperiod: 10 pp in Argentina; around 4 pp in Brazil, Costa Rica and Mexico; 2 pp in Paraguay and 1 pp in Peru. According to ILO (2020b), this shows, on one hand, that the increase in the level of economic activity did not necessarily entail the creation of new formal occupations, but rather that firms resorted to raising the working hours to face the growing demand in production, or workers that were temporarily suspended or absent returned. On the other hand, own-account workers, many of whom are informal, had the chance to go back to activities that had been suspended by the lockdown. The increase in informal waged occupations can also be related, to some extent, with the reopening of small businesses, which have a higher informality rate. Despite the increase in informal positions, the rate of informality in the third quarter of 2021 was similar to pre-pandemic values. The exception was Peru, at 2 pp higher.^5^

To sum up, the dynamic of labour informality is crucial to understanding the significant and heterogeneous impacts of the crisis on overall employment. Even more so considering that informal employment represents a significant proportion of total employment in all of these countries: about 40% in Costa Rica and Brazil, 44% in Argentina, 54% in Mexico, 63% in Paraguay and 73% in Peru.^6^

In the following section, we deepen the analysis by incorporating the study of labour market flows. First, we analyse transition matrices across labour statuses. Then, the focus is on entry rates and exit rates from both formal and informal occupations. We include a gender perspective when comparing men’s probabilities of exiting from informal occupations with those of women. Finally, we analyse the labour destinations of those who leave an informal occupation.

## Results from the dynamic analysis and discussion

### Transition matrices across different labour statuses

Transition matrices were calculated for three different subperiods: (1) before the outbreak of the pandemic; (2) when labour market impact was at its peak; (3) during the partial recovery of employment (Table 4). The values on the diagonal of these matrices are the retention rate in the initial labour status. Complementary, the values outside the diagonal show transits between the three labour statuses (employment, unemployment and inactivity).

**Table 4:** Transition matrices across different labour statuses

Argentina	I19–II19				I20–II20				II21–III21			
	E	U	I	Total	E	U	I	Total	E	U	I	Total
E	48	2	4	54	41	3	10	54	51	1	4	56
U	2	2	1	6	1	2	3	6	3	2	1	6
I	4	2	35	40	2	1	36	40	4	2	33	38
Total	54	6	40	100	44	6	50	100	57	5	38	100

Brazil	I19–II19				I20–II20				II21-III21			
	E	U	I	Total	E	U	I	Total	E	U	I	Total
E	50	2	3	55	48	2	5	55	48	1	1	51
U	2	3	2	8	1	4	2	7	2	6	1	8
I	4	2	32	38	1	1	36	38	2	1	37	41
Total	55	7	37	100	50	7	42	100	52	8	41	100

Costa Rica	I19–II19				I20–II20				II21-III21			
	E	U	I	Total	E	U	I	Total	E	U	I	Total
E	49	2	4	55	40	8	6	54	43	3	3	49
U	3	3	2	7	1	3	3	8	4	4	2	10
I	3	3	32	38	2	2	34	38	3	2	55	41
Total	55	7	38	100	43	14	43	100	50	10	40	100

Mexico	I19–II19				I20–II20				II21–III21			
	E	U	I	Total	E	U	I	Total	E	U	I	Total
E	50	1	6	57	46	1	12	59	49	1	7	57
U	1	0	1	2	1	0	1	2	1	0	1	2
I	7	1	33	41	5	0	34	39	7	1	33	41
Total	58	2	40	100	52	2	46	100	57	2	41	100

Paraguay	I19–I20				II19–II20				III20–III21			
	E	U	I	Total	E	U	I	Total	E	U	I	Total
E	58	3	7	68	56	3	10	69	57	2	6	65
U	3	1	1	5	2	1	1	4	4	1	1	6
I	6	2	19	27	5	1	21	27	9	2	18	29
Total	67	5	28	100	63	5	32	100	69	5	26	100

Peru	I19–I20				II19–II20				III20–III21			
	E	U	I	Total	E	U	I	Total	E	U	I	Total
E	53	2	8	63	28	4	33	65	40	2	4	46
U	3	1	2	6	1	1	3	5	6	2	2	11
I	7	2	21	31	2	1	27	30	11	4	28	43
Total	63	6	31	100	31	6	63	100	57	8	34	100

E: Employment; U: unemployment; I: inactive

Source: Own elaboration based on household surveys

Before the pandemic, between 50 and 60% of working-age people were employed, either between two consecutive quarters (Argentina, Brazil, Costa Rica and Mexico) or during the same quarter of two consecutive years (Paraguay and Peru). However, in line with the aforementioned marked reduction in employment during the first half of 2020, job retention fell. The intensity of fall was different across countries. Interestingly, the ranking of countries according to the magnitude of the contraction in total employment is perfectly repeated here. The drop in job retention was about − 2 pp in Paraguay and Brazil, − 4 pp Mexico, and it reached − 7 pp in Argentina, − 9 pp in Costa Rica and − 25 pp in Peru (Table 4).

The increase in exit rates from occupations, however, was not the only driver of the contraction in total employment. As the table shows, this occurred along with decreasing entry flows into occupations, especially from outside the labour force. While both types of transitions played a role in the fall in occupations during the first half of 2020, except for Brazil, the increase in exit rates from employment accounted for the total job destruction majority (60% to 80%) (Table 5). This is an expected outcome, considering the severity and nature of the economic crisis, resulting initially from a supply shock caused by the immediate shutdown of non-essential economic activities, and then, from a negative demand shock arising from the large reduction in labour and family incomes.

**Table 5:** Contribution of entry and exit flows to employment variation

Country	Percentage of employment contraction (IV2019–II2020) explained by:			Percentage of employment recovery (II2020–III2021) explained by:		
	Increase in exit rate	Contraction in entry rate	Total	Contraction in exit rate	Increase in entry rate	Total
Argentina	72	28	100	73	27	100
Brazil	32	68	100	76	24	100
Costa Rica	78	22	100	68	32	100
Mexico	74	26	100	69	31	100
Paraguay	56	44	100	42	58	100
Peru	77	23	100	66	34	100

Source: Own elaboration based on household surveys

As previously mentioned, the decrease in employment also came along with a sharp fall in labour force participation. The results from the transition matrices (Table 4) indicate that the latter was due, on one hand, to individuals remaining longer outside the labour force during the first phase of the crisis; on the other hand, to a greater extent, more intense flows from employment, and in some cases unemployment, into inactivity. The increase in outflows from the labour force was between 4 and 7 pp in Brazil, Costa Rica, Mexico and Paraguay, 10 pp in Argentina and about 30 pp in Peru.

Symmetrically, the partial recovery of jobs as from mid-2020 was explained principally by a drop in the job exit rate (Table 5). In other words, this process was driven by greater stability in existing occupations rather than faster creation of new occupations (except in Paraguay). In a way, this was an expected result at the beginning of the road to economic recovery, especially considering the high levels of health and economic uncertainty that still prevailed at the time. Moreover, this finding is in line with the observation that working hours reacted to the economic recovery faster than aggregate employment (ILO 2021b).

In the comparison of transition matrices in 2019 and 2021, Argentina stands out as the only country with a higher employment retention rate. This is consistent with the fact that, as mentioned, the Argentine employment rate in 2021 surpassed its pre-pandemic value. At the opposite end, Costa Rica and Peru had lower retention rates, 6 pp and 13 pp respectively (Table 4). As shown earlier, by the end of 2021 these two countries exhibited the largest employment gaps in comparison with 2019.

### Labour flows associated with formality and informality

**Fig. 1:**
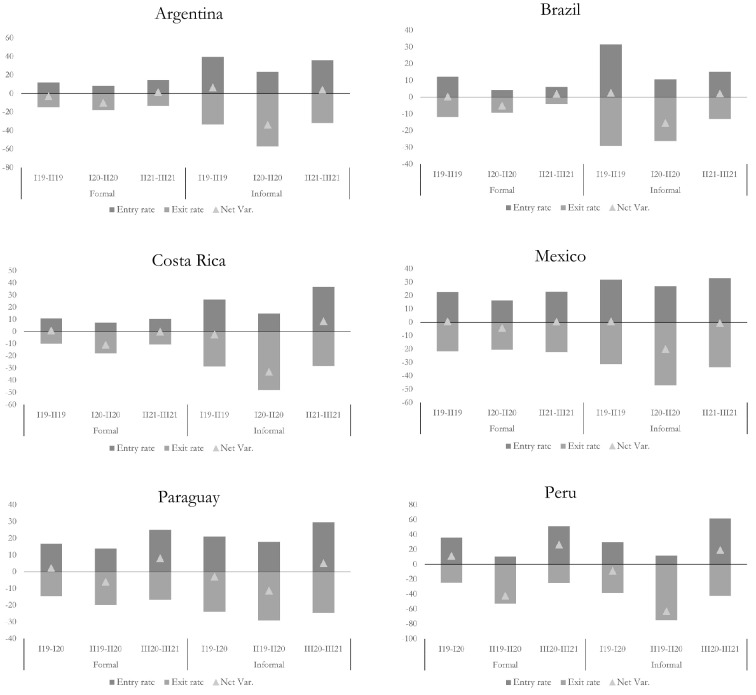
Exit and entry rates for informal and formal occupation, and net employment variation. Source: Own elaboration based on household surveys

Behind the global overview, significant differences appear in the labour flows of formal and informal workers (Fig. 1). Both before and during the pandemic, exit and entry rates were significantly higher in informal occupations than in formal ones, (except for Peru which had entry rates to formal occupations in 2019 that exceeded those for informal positions). These results are consistent with the abovementioned evidence showing higher labour turnover in informality in Latin America (Beccaria et al 2015; Maurizio et al. 2021). Exit rates for both informal and formal occupations increased significantly in 2020, except in Brazil. In Argentina, Costa Rica, Mexico and Peru this rise was even more intense (in percentage points) for informal jobs. The increase in exit rates of informal (formal) workers was around 19 pp (8 pp) in Costa Rica, 24 pp (3 pp)^7^ in Argentina, and 36 pp (28 pp) in Peru. In Paraguay the rise was similar in both cases (around 5 pp).

**Fig. 2:**
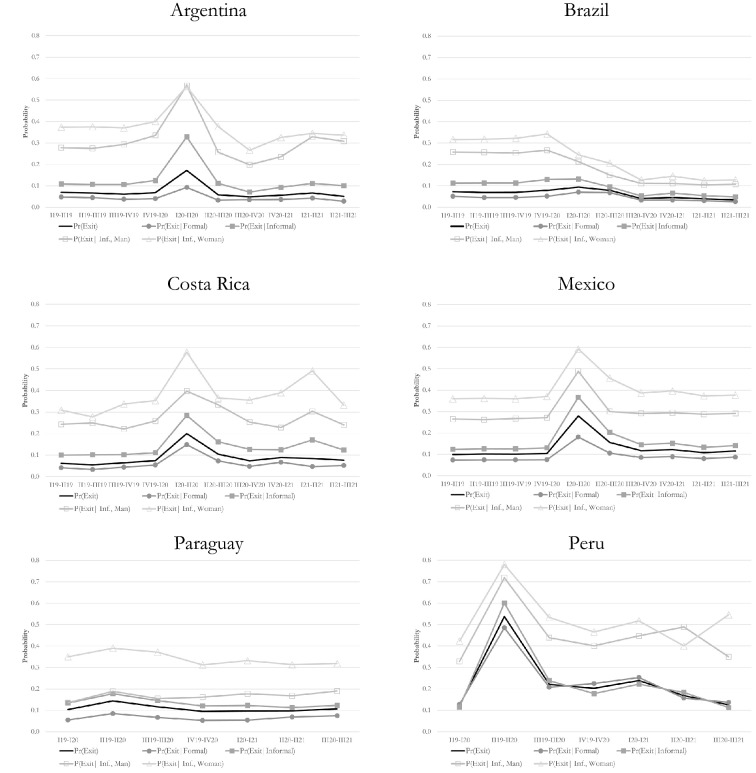
Probabilities to exit occupation by formal/informal status and by gender. Covariates of logit model: **a** to estimate the probability to exit from a job: age, education, gender, head of household, married, children in household, region, branch of activity, size firm, panel-period, informal, interactions between last two dummies. **b** To estimate the probability to exit form an informal job: age, education, head of household, married, children in household, region, branch of activity, size firm, panel-period, gender, interactions between last two dummies. (*) 95% confidence intervals are presented between brackets. Source: Own elaboration based on household surveys

These differences in labour dynamics between both groups of workers could be related to personal characteristics or other attributes of their jobs (in addition to informality). To evaluate the independent role of each of these factors, logit models were run, separately for each country.^8^ The results from these regressions are shown in Table 6 and the predicted probabilities for every panel-period holding the rest of covariates at their means, in Fig. 2.^9^ Three main conclusions are drawn from them. First, the results confirm that -after controlling for other variables- the exit rates from an occupation are significantly higher among informal workers than among formal workers; i.e. the gap in exit rate between informal and formal employment is always positive and statistically significant, except in Peru^10^ (Table 6).

**Table 6:** Employment exit probability

	Argentina	Brazil	Costa Rica	Mexico	Paraguay	Peru
Informality	0.883***	0.868***	0.946***	0.530***	0.979***	− 0.119
Panel (base 1Q19-2Q19): 1Q20-2Q20	0.710***	0.366***	1.392***	0.655***	–	–
Interaction: 1Q20-2Q20*Informality	0.679***	− 0.172***	− 0.126	0.271**	–	–
Panel (base 1Q19-1Q20): 2Q19-2Q20	–	–	–	–	0.469**	1.857***
Interaction: 2Q19-2Q20*Informality	–	–	–	–	− 0.134	0.585***

*** p < 0.01, **p < 0.050, * p < 0.1. Covariates: age, education, gender, household position, marital status, presence of children in household, region, branch of activity, size of the firm, informality, period, and interaction terms between informality and period, Number of observations: Argentina = 9198, Brazil = 57,624, Costa Rica = 8079, Mexico = 137,403, Paraguay = 3672, Peru = 2934

Source: Own elaboration based on household surveys

Figure 2 allows us to compute the magnitude of the instability gap between formal and informal workers. Before the pandemic, in Argentina, Brazil, and Costa Rica, the probability of leaving employment between two consecutive quarters was double for informal workers (around 10%) compared to formal workers (around 5%). In Peru both probabilities were quite similar, at around 13%. In Mexico they were about 13% for formal and 8% for informal workers. The instability gap was somewhat higher in Paraguay (13% and 5%, respectively).

Second, also confirming the descriptive results, the exit rates from any occupation increased in all of the countries during the first half of 2020 (Table 6). For instance, the probability of leaving employment rose 20% in Brazil, 40% in Paraguay, 150% in Argentina, almost 170% in Costa Rica and Mexico, and 340% in Peru (Fig. 2). Third, and even more important for the purpose of this study, econometric results confirm that in Argentina, Mexico and Peru, the jump in the exit rate from employment during the peak of the crisis was significantly higher among informal workers than formal workers (Table 6). In Argentina, while the exit rate of formal employment doubled, that of informal employment almost tripled. In México the first rate increased 76% while the second rate doubled. In Peru, the exit rate from formality increased almost 4 times while from informality it grew by a little more than 5 times (Fig. 2). In Brazil the instability gap between informal and formal occupations slightly reduced. Finally, in line with previous results, Costa Rica and Paraguay did not register statistically significant differences between both groups of workers (Table 6).

Several factors account for the more intense increase in exit rates from informal occupation in the countries where this occurred. First, the greater rate of informality among certain sectors of activity—such as domestic services, restaurants, construction—that had to stop their activities due to not being considered “essential” (ILO 2020b). Second, it is easier to terminate an informal wage contract and this type of job is more prevalent in smaller companies, which found it more difficult to endure such a huge economic crisis. Additionally, many informal self-employed workers were not exempted from social distancing and for most of them, the possibilities of teleworking were limited. Maurizio (2021) points out that the rise in teleworking during the pandemic in Latin America was observed almost exclusively among formal salaried workers.

The higher stability of salaried formal occupations, in turn, could indicate that employers believed the contraction phase would be short-lived. Firms worked out strategies, such as the reduction of the working day or temporary labour contract suspension, which made continued formal employment possible (ILO 2020b; Beccaria et al. 2022). Finally, the public policies supporting salaried formal employment, put in place in some of these countries, could be another factor associated with these divergent dynamics between formal and informal occupations (ILO 2021b; Beccaria et al 2022).^11^ In 2020, Argentina implemented an emergency assistance programme to support formal employment (*Programa de Asistencia de Emergencia al Trabajo y la Producción*–ATP), cutting down on employers’ social security contributions by 95% and providing payroll subsidies. It also prohibited unfair dismissals, and dismissals due to lack of (or reductions in) work. In Peru private-sector employers received a subsidy of up to 35% of the gross monthly salaries of their employees. In Brazil and Paraguay, where the increase in the instability gap between informal and formal workers was not proven by econometric results, policies to support formal employment were also implemented. The Brazilian emergency employment and income protection programme (*Benefício Emergencial de Preservação do Emprego e da Renda*) protected the income of formal wage earners on furlough or whose working hours or wages had been reduced. Paraguay established a subsidy for formal wage workers earning up to double the minimum wage and whose job contracts were suspended due to the cessation of economic activities caused by the pandemic.

Together with the rise in exit rates from occupations, the diminished intensity of entry flows into both formal and informal occupations also accounted for the contraction in employment during the second quarter of 2020. However, in the case of informal workers, as expected, the increased exit rate was the main driver of the employment drop, apart from in Brazil. The same was observed among formal workers in Costa Rica, Paraguay and Peru, while in Argentina and México both types of labour flows had similar intensity (Table 7).^12^

**Table 7:** Contribution of entry and exit flows to the evolution of employment

Country	Percentage of employment in the contraction phase explained by:				Percentage of employment in the recovery phase explained by:			
	Increase in exit rate		Contraction in entry rate		Contraction in exit rate		Increase in entry rate	
	Formal	Informal	Formal	Informal	Formal	Informal	Formal	Informal
Argentina	48	61	52	39	42	62	58	38
Brazil	− 43	− 22	143	122	80	78	20	22
Costa Rica	64	66	36	34	74	63	26	37
Mexico	49	58	51	42	29	58	71	42
Paraguay	82	62	18	38	31	36	69	64
Peru	57	68	43	32	53	65	47	35

Source: Own elaboration based on household surveys

Econometric results confirm that during the recovery phase there was a significant reduction in exit rates from both types of occupations in all six countries. This meant that in Argentina, Brazil, Costa Rica and Peru these exit rates returned to the same (or even lower) pre-pandemic levels (Fig. 2). An increase in inflows into these occupations was also observed during this period (Table 7). As we will see later, this is explained by the return to employment—in particular, informal employment—of those workers who left the labour force during the first phase of the crisis.

To sum up, at the crisis’ peak, total employment significantly fell, mostly led by the rise in exit rates from informal occupations. Symmetrically, the partial recovery in employment was driven by informal employment, mainly due to the reduction in its exit rate intensity. Therefore, the following two sections analyse in more detail the exit rates from informal occupations.

### Exit rates from informal occupation by gender

As described before, in Latin America women experienced a greater contraction in employment (and in economic participation) than men (ILO 2021b; ECLAC 2021b). This was in line with what was observed worldwide (ILO 2021; Alon et al. 2020). This previous literature found several factors explaining this result. This section assesses whether, in addition to these factors, women were more likely than men to exit from informal employment during the contraction phase. To that end, logit models were run for each country, and the results are presented in Table 8.^13^

**Table 8:** Probability to exit from an informal occupation by gender

	Argentina	Brazil	Costa Rica	Mexico	Paraguay	Peru
Female	0.436***	0.291***	0.328**	0.473***	1.234***	0.404**
Panel (base 1Q19–2Q19): 1Q20–2Q20	1.227***	− 0.352***	0.715***	0.701***	–	–
Interaction: 1Q20–2Q20*Female	− 0.457***	− 0.113**	0.406**	0.108	–	–
Panel (base 1Q19-1Q20): 2Q19–2Q20					0.171*	1.576***
Interaction: 2Q19–2Q20*Female					− 0.225	− 0.075

*** p < 0.01, **p < 0.050, * p < 0.1. Covariates: age, education, gender household position, marital status, presence of children in household, region, branch of activity, size of the firm, period, and interactions between periods and gender. Number of observations: Argentina = 27,395, Brazil = 250,412, Costa Rica = 24,025, Mexico = 353,442, Paraguay = 15,070, Peru = 5939

Source: Own elaboration based on household surveys

First, the coefficients of the covariate “female” were positive and statistically significant in the six countries, meaning that the probability to exit an informal occupation throughout the whole period was higher for women than for men. Second, the coefficients of the covariate "panel" were also positive and statistically significant, except for Brazil. This result confirms that in the remaining five countries, there was a significant rise in occupational instability for informal workers during the contraction phase of the crisis. Third, the interaction effect between female and panel -the covariate of most interest in these regressions—was positive and statistically significant only in Costa Rica. This implies that in this country alone, the jump in exit rates from informal employment was more intense for women than for men. In Argentina and Brazil this coefficient was also statistically significant but negative. As shown in Fig. 2, in Brazil this is explained by the fact that during the contraction phase the exit rate of informally employed men dropped by 20% while the exit rate for women reduced even more, 28%. In Argentina, both rates increased but with high intensity among men. Finally, in Mexico, Paraguay and Peru this coefficient was not statistically significant (Table 8).

In summary, except for Costa Rica, the greatest loss of female occupations during the first half of 2020 does not originate from a more intense increase in female exit rates from informal employment in comparison with those of men. Rather, the huge reduction in female employment can be explained by a “composition effect” associated with the higher informality incidence among women and higher female employment in the economic sectors which were most affected by the crisis. Growing difficulties among women to remain in the labour force given the closure of care services and schools, is another potential factor that may explain the worse female labour performance during this period.

### Labour destinations after exiting informal occupation

**Fig. 3:**
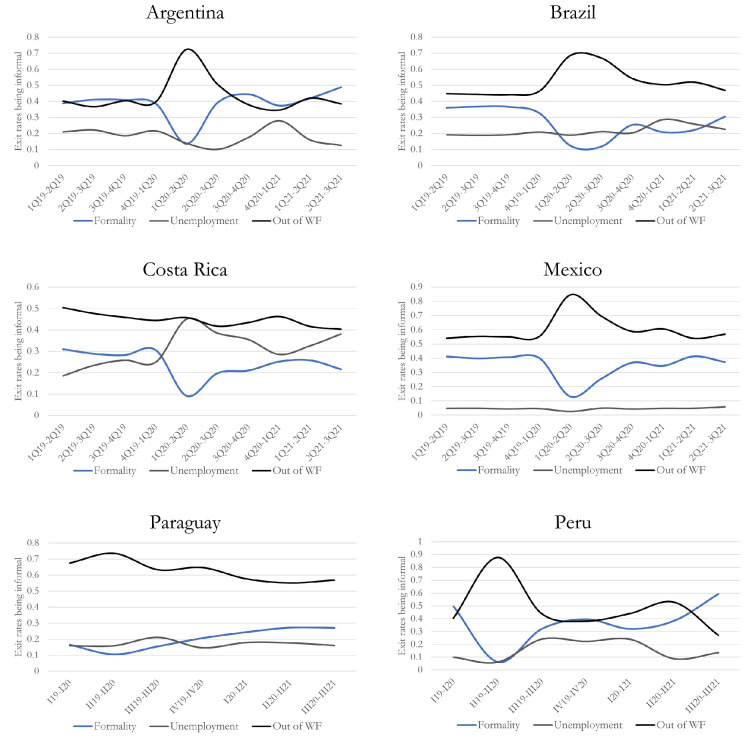
Conditional transition probabilities after leaving an informal occupation. Covariates: age, education, gender, household position, marital status, presence of children in household, region, branch of activity, size of the firm and period. Number of observations: Argentina = 9198, Brazil = 57,624, Costa Rica = 8079, Mexico = 137,403, Paraguay = 3672, Peru = 2934. Source: Own elaboration based on household surveys

In addition to the estimate of exit rate intensity, the identification of the destinations reached by workers after leaving an informal job is another relevant issue in this context. Multinomial logit models were run to assess the conditional probability to transit to a formal occupation, to unemployment or inactivity. Figure 3 shows the predicted margins from these regressions. In line with the descriptive findings, the jump in exit rates from informal occupations resulted in a significant increase in labour force outflows in early 2020 (except for Costa Rica); in fact, inactivity was the most likely destination. The probability of going into inactivity after leaving informality was about 68% in Brazil, more than 20 percentage points higher than before the pandemic. In Argentina this rate was 73%, which represents a jump of about 30 percentage points. In Paraguay, Mexico and Peru, this destination also breaks records with rates of 74%, 80% and almost 90%, respectively.

As expected, transitions from informal occupations to formal ones significantly dropped during the most critical phase of this crisis. This is consistent with the fall in entry rates to formal occupations as analysed above. In 2019, around 30% to 40% of informal workers who left their occupations obtained formal occupation in Argentina, Brazil, Costa Rica, Mexico and Peru; that figure was about 20% in Paraguay. All of these ratios plummeted at the beginning of the pandemic. In Mexico, for instance, the fall in this probability between 2019 and 2020 was about 28 pp. Even more intense was the contraction in Peru, about 43 pp. In the remaining countries the reduction was less intense but also significant.

Once the peak of the crisis passed, the intensity of outflows from the labour force decreased while transits towards formal occupations began to (slowly) recover. However, when comparing the structure of destinations in 2019 with that in 2021, we can observe that in Brazil, Costa Rica and Mexico, transitions to formality after leaving informality were still significantly less intense than before the pandemic.

## Robustness check: attrition bias correction

As previously stated, the presence of non-random attrition could only be assessed in Brazil, Mexico, and Paraguay. Two different tests were used, as detailed in the Appendix. The first test was that proposed by Fitzgerald et al. (1998), where the pseudo R squared is interpreted as the contribution of systematic attrition to the dropout probability. The second test was proposed by Becketti et al. (1988)—BGLW—to evaluate if the attrition is related to the labour status. Additional file 4: Table S2 shows these results.

The first test confirmed the randomness of attrition in Brazil, since the pseudo R squared was small along the periods (between 0.4% and 1.7%). The BGLW test shows that none of the coefficients associated with the impact of attrition on the labour status were statistically significant. Consequently, no sample adjustment to correct for attrition should be applied in this country. The portion of attrition that could be explained was greater in Mexico than in Brazil, although it remained low, ranging between 1% and 2.5%. However, the BGLW test concluded that the impact of attrition on the labour status was significant, thus sample adjustment applies. Finally, Paraguay also showed very low pseudo R squared, ranging between 0.1% and 0.2%. The result of the global test confirmed that systematic attrition was not significant along the panels under study. However, in the case of the BGLW tests, except for the first and the last panel, the impact of attrition on the labour status variable was statistically significant.

Therefore, due to the evidence of the impact of attrition on the labour status variable in Mexico and Paraguay, a sample reweighting correction was applied. Probit regression models were run to estimate the probability to drop out of the sample, controlling for observable characteristics. Then, reweighting factors were built—based on the estimated probabilities- and calibrated to the size of the population projected in each panel. Additional file 5: Table S3 shows the transition matrices corresponding to these two countries with and without the reweighting adjustment. This correction does not significantly modify the results in these two countries, suggesting that the prior findings remain and are robust.

## Final remarks

During 2020, Latin America faced an unprecedented economic and labour crisis because of the COVID-19 pandemic. This particular context led to adjustments in the labour market that were different from those observed in previous crises.

This paper analysed, from a dynamic and comparative perspective, labour transits triggered by the pandemic in six Latin American countries: Argentina, Brazil, Costa Rica, Mexico, Paraguay and Peru. This analysis contributes to a better understanding of the effects of the pandemic on labour markets in Latin America by providing insights into the intensity and direction of labour flows during this exceptional time.

The results showed that, unlike previous crises, during the contraction phase labour informality did not play the traditional counter-cyclical role, but rather intensified the overall contraction in employment by dropping more intensely than in formal occupations. This was, in turn, mostly led by a significant rise in exit rates from these jobs, rather than by a decrease in the entry rates. Most informal workers who lost their jobs transitioned out of the labour force. Counter to this, transits from informal to formal jobs significantly dropped during the most critical phase of this crisis. However, the partial recovery in employment since mid-2020 was led by a rise in this type of occupation. The drop in the intensity of exit rates from these occupations mostly explains this behaviour, while a greater intensity in entry flows to informality from inactivity also accounts for this trend. As a result, in the third quarter of 2021, almost two years after the onset of the pandemic in Latin America, informality was extremely high in all studied countries, ranging from 39 to 73% of total employment.

Given this critical context, it is crucial to maintain the policies that were applied in Latin America during 2020 and 2021, while also adopting a broader agenda of far-reaching and comprehensive policies. Policies should be implemented not only to preserve current formal employment, but also to promote the creation of new formal jobs in the region. Moreover, considering the negative impacts of high occupational turnover on income instability among informal workers, it is essential to strengthen income support policies as well as moving towards long-lasting social protection schemes which reach the most vulnerable people.

### Supplementary Information

**Additional file 1: Fig. S1.** Evolution of the total employment, formal employment, informal employment and informality rate.

**Additional file 2: Fig. S2.** Contribution of formal and informal employment to total labour contraction and partial labour recovery.

**Additional file 3: Table S1.** Empirical identification of informality.

**Additional file 4: Table S2.** Testing for random attrition.

**Additional file 5: Table S3.** Reweighted and not-reweighted transition matrices to correct attrition bias.

## Data Availability

The datasets supporting the conclusions of this article are available in the following public domains:

**Table:** 

Country	Period	Link URL
Argentina	I Q 2019	https://www.indec.gob.ar/ftp/cuadros/menusuperior/eph/EPH_usu_1_Trim_2019_txt.zip
	II Q 2019	https://www.indec.gob.ar/ftp/cuadros/menusuperior/eph/EPH_usu_2_Trim_2019_txt.zip
	III Q 2019	https://www.indec.gob.ar/ftp/cuadros/menusuperior/eph/EPH_usu_3_Trim_2019_txt.zip
	IV Q2019	https://www.indec.gob.ar/ftp/cuadros/menusuperior/eph/EPH_usu_4_Trim_2019_txt.zip
	I Q 2020	https://www.indec.gob.ar/ftp/cuadros/menusuperior/eph/EPH_usu_1_Trim_2020_txt.zip
	II Q 2020	https://www.indec.gob.ar/ftp/cuadros/menusuperior/eph/EPH_usu_2_Trim_2020_txt.zip
	III Q 2020	https://www.indec.gob.ar/ftp/cuadros/menusuperior/eph/EPH_usu_3_Trim_2020_txt.zip
	IV Q2020	https://www.indec.gob.ar/ftp/cuadros/menusuperior/eph/EPH_usu_4_Trim_2020_txt.zip
	I Q 2021	https://www.indec.gob.ar/ftp/cuadros/menusuperior/eph/EPH_usu_1_Trim_2021_txt.zip
	II Q 2021	https://www.indec.gob.ar/ftp/cuadros/menusuperior/eph/EPH_usu_2_Trim_2021_txt.zip
	III Q 2021	https://www.indec.gob.ar/ftp/cuadros/menusuperior/eph/EPH_usu_3_Trim_2021_txt.zip
Brazil	I Q 2019	https://www.ibge.gov.br/estatisticas/downloads-estatisticas.html?caminho=Trabalho_e_Rendimento/Pesquisa_Nacional_por_Amostra_de_Domicilios_continua/Trimestral/Microdados/2019
	II Q 2019	
	III Q 2019	
	IV Q2019	
	I Q 2020	https://www.ibge.gov.br/estatisticas/downloads-estatisticas.html?caminho=Trabalho_e_Rendimento/Pesquisa_Nacional_por_Amostra_de_Domicilios_continua/Trimestral/Microdados/2020
	II Q 2020	
	III Q 2020	
	IV Q2020	
	I Q 2021	https://www.ibge.gov.br/estatisticas/downloads-estatisticas.html?caminho=Trabalho_e_Rendimento/Pesquisa_Nacional_por_Amostra_de_Domicilios_continua/Trimestral/Microdados/2021
	II Q 2021	
	III Q 2021	
Costa Rica	I Q 2019	http://sistemas.inec.cr/pad5/index.php/catalog/246
	II Q 2019	
	III Q 2019	
	IV Q2019	
	I Q 2020	http://sistemas.inec.cr/pad5/index.php/catalog/276
	II Q 2020	
	III Q 2020	
	IV Q2020	
	I Q 2021	http://sistemas.inec.cr/pad5/index.php/catalog/284
	II Q 2021	
	III Q 2021	
Mexico	I Q 2019	https://www.inegi.org.mx/contenidos/programas/enoe/15ymas/microdatos/2019trim1_dbf.zip
	II Q 2019	https://www.inegi.org.mx/contenidos/programas/enoe/15ymas/microdatos/2019trim2_dbf.zip
	III Q 2019	https://www.inegi.org.mx/contenidos/programas/enoe/15ymas/microdatos/2019trim3_dbf.zip
	IV Q2019	https://www.inegi.org.mx/contenidos/programas/enoe/15ymas/microdatos/2019trim4_dbf.zip
	I Q 2020	https://www.inegi.org.mx/contenidos/programas/enoe/15ymas/microdatos/2020trim1_dbf.zip
	II Q 2020	https://www.inegi.org.mx/contenidos/investigacion/etoe/microdatos/etoe_2020_abril_cpv2020_dbf.zip https://www.inegi.org.mx/contenidos/investigacion/etoe/microdatos/etoe_2020_mayo_cpv2020_dbf.zip https://www.inegi.org.mx/contenidos/investigacion/etoe/microdatos/etoe_2020_junio_cpv2020_dbf.zip
	III Q 2020	https://www.inegi.org.mx/contenidos/programas/enoe/15ymas/microdatos/enoe_n_2020_trim3_dbf.zip
	IV Q2020	https://www.inegi.org.mx/contenidos/programas/enoe/15ymas/microdatos/enoe_n_2020_trim4_dbf.zip
	I Q 2021	https://www.inegi.org.mx/contenidos/programas/enoe/15ymas/microdatos/enoe_n_2021_trim1_dbf.zip
	II Q 2021	https://www.inegi.org.mx/contenidos/programas/enoe/15ymas/microdatos/enoe_n_2021_trim2_dbf.zip
	III Q 2021	https://www.inegi.org.mx/contenidos/programas/enoe/15ymas/microdatos/enoe_n_2021_trim3_dbf.zip
Paraguay	I Q 2019	https://www.ine.gov.py/microdatos/register/EPHC/EPHC-2019/Primer%20Trimestre/REG02_EPHC_T1_2019.SAV
	II Q 2019	https://www.ine.gov.py/microdatos/register/EPHC/EPHC-2019/Segundo%20Trimestre/REG02_EPHC_T2_2019.SAV
	III Q 2019	https://www.ine.gov.py/microdatos/register/EPHC/EPHC-2019/Tercer%20Trimestre/REG02_EPHC_T3_2019.SAV
	IV Q2019	https://www.ine.gov.py/microdatos/register/EPHC/EPHC-2019/Cuarto%20Trimestre/REG02_EPHC_T4_2019.SAV
	I Q 2020	https://www.ine.gov.py/microdatos/register/EPHC/EPHC-2020/Primer%20Trimestre/REG02_EPHC_T1_2020.SAV.SAV
	II Q 2020	https://www.ine.gov.py/microdatos/register/EPHC/EPHC-2020/Segundo%20Trimestre/REG02_EPHC_T2_2020.SAV
	III Q 2020	https://www.ine.gov.py/microdatos/register/EPHC/EPHC-2020/Tercer%20Trimestre/REG02_EPHC_T3-2020.SAV
	IV Q2020	https://www.ine.gov.py/microdatos/register/EPHC/EPHC-2020/Cuarto%20Trimestre/REG02_EPHC_T4-2020.SAV
	I Q 2021	https://www.ine.gov.py/microdatos/register/EPHC/EPHC-2021/Primer%20Trimestre/REG02%20EPHC_T1-2021.SAV
	II Q 2021	https://www.ine.gov.py/microdatos/register/EPHC/EPHC-2021/Segundo%20Trimestre/REG02_EPHC_2do%20Trim%202021.SAV
	III Q 2021	https://www.ine.gov.py/microdatos/register/EPHC/EPHC-2021/Tercer%20Trimestre/REG02_EPHC_3er%20Trim%202021.SAV
Peru	I Q 2019	http://iinei.inei.gob.pe/iinei/srienaho/descarga/DBF/636-Modulo76.zip
	II Q 2019	http://iinei.inei.gob.pe/iinei/srienaho/descarga/DBF/643-Modulo76.zip
	III Q 2019	http://iinei.inei.gob.pe/iinei/srienaho/descarga/DBF/648-Modulo76.zip
	IV Q2019	http://iinei.inei.gob.pe/iinei/srienaho/descarga/DBF/655-Modulo76.zip
	I Q 2020	http://iinei.inei.gob.pe/iinei/srienaho/descarga/DBF/688-Modulo76.zip
	II Q 2020	http://iinei.inei.gob.pe/iinei/srienaho/descarga/DBF/695-Modulo76.zip
	III Q 2020	http://iinei.inei.gob.pe/iinei/srienaho/descarga/DBF/700-Modulo76.zip
	IV Q2020	http://iinei.inei.gob.pe/iinei/srienaho/descarga/DBF/727-Modulo76.zip
	I Q 2021	http://iinei.inei.gob.pe/iinei/srienaho/descarga/CSV/735-Modulo76.zip
	II Q 2021	http://iinei.inei.gob.pe/iinei/srienaho/descarga/CSV/742-Modulo76.zip
	III Q 2021	http://iinei.inei.gob.pe/iinei/srienaho/descarga/CSV/747-Modulo76.zip https://www.inegi.org.mx/contenidos/investigacion/etoe/microdatos/etoe_2020_abril_cpv2020_dbf.zip https://www.inegi.org.mx/contenidos/investigacion/etoe/microdatos/etoe_2020_mayo_cpv2020_dbf.zip https://www.inegi.org.mx/contenidos/investigacion/etoe/microdatos/etoe_2020_junio_cpv2020_dbf.zip

## Appendix

### Attrition bias identification and correction

The strategy used to identify and correct the presence of attrition is composed by three stages which are detailed below.

### Identification of potential participant dropout

Attrition was identified by comparing the final number of interviews per person with the number of visits required according to the rotation scheme. If the number of the last visit is below the required number, this means that the person dropped out of the sample early, thus evidencing the presence of attrition.

### Sample random attrition

Attrition can be random or systematic. When systematic, it can generate biased estimations. To evaluate whether attrition is random or not, we followed the strategy proposed by Fitzgerald et al. (1998). The test is based on the results of the probability to drop out of the sample. A probit regression model is conducted, where the dependent variable is an indicator of panel drop-out and the covariates are observable characteristics associated with this phenomenon: age, education, gender, household position, marital status, labour status, region, presence of children and elderly people in the household, and interactions of these variables with gender. The visit number is also included to control for a potential increase in the probability to drop out in the following interviews. After running the probit model, the pseudo-R-squared can be interpreted as the proportion of attrition explained by observable characteristics. Complementary to these statistics, a Wald test of global significance is performed. If this is statistically significant, a sample correction applies.

In addition, the Becketti et al. (1988) (BGLW) strategy was used to test if attrition has any impact on the variable used to build the transition matrices. To that end, a regression model was conducted where the dependent variable was the “labour status” (employed, unemployed or out of the labour force) at the initial wave. The covariates used correspond to the characteristics of the individual, the household, and the region. In addition, a dummy variable to identify those individuals dropping out of the panel earlier than expected according to the rotating scheme, was included. Interactions between this variable and the other covariates were also included. A Wald test for global statistical significance of this dummy variable and its interaction terms was estimated to determine if attrition does or does not generate selection-bias regarding the statistics related to labour status.

### Sample reweighting and calibration

Finally, if attrition was not random the estimations had to be corrected. The methodology proposed by Rosemabaun and Rubin (1984) and by Wooldridge (2002) is used to reweight the sample in order to correct the bias. This method consists of using one or more auxiliary variables that affect both the probability of attrition and the variable of interest. Particularly, the method requires the estimation of inverse probability calculated from a probit or logit model. If A* is the attrition variable, then:

9 $${A}^{*}={x}_{i1}\gamma + {z}_{i1}\delta +{\mu }_{i}$$

where A* takes value one if the individual drops out of the sample and zero in other cases, xi1 is a vector of individual characteristics and ${z}_{i1}$ is a vector of auxiliary variables correlated with the probability of leaving the panel, both observed in the first panel.

Then a logistic regression model is used to estimate the attrition probabilities ${p}_{i}^{A*}$

10 $$p_{i}^{A*}=P(A*=1|X,~Z)=\frac{{{e}^{{\gamma }^{\prime} X++\delta ^{\prime} Z}}}{1+{{e}^{{\gamma }^{\prime} X++\delta ^{\prime} Z}}}$$

The next step is to calculate the inverse probability to adjust initial sample weights:

11 $${W}_{i}^{*}= \frac{{W}_{i}}{{(1-p}_{i}^{A*})}$$

where w_i_ are the original weights. Finally, a global adjustment is made to calibrate the data of the sample and scale it to the original size of population.

From this formula, it is easily understood that, in order to correct the bias attrition, a greater weight will be assigned to individuals with similar characteristics to those that drop out of the sample early.

## Footnotes

1 The percentage change in employment in response to the percentage change in the level of activity.

2 See Hamilton (1994) for a concise explanation of Markov chains.

3 In addition, entry rates are calculated as the proportion of outflows from* j* to* i* over N_i_.

4 The Oxford Stringency Index, calculated by the Oxford Coronavirus Government Response Tracker (OxCGRT), is a composite measure of nine different response metrics. These include: “school closures, workplace closures, cancellation of public events, restrictions on public gatherings, closure of public transport, stay-at-home requirements; public information campaigns, restrictions on internal movements and international travel controls (Our World in Data 2022).

5 Although employment recovery was not accompanied by greater informality, the countries that still show significant differences in the volume of occupations in comparison with 2019, could close their gap with higher informality rates than those observed in that year. The long-term impact of the crisis on these countries, however, deserves a detailed analysis, which goes beyond the scope of this study.

6 These figures correspond to the III quarter of 2021.

7 Even though in Costa Rica the increase in the exit rate from informality was more intense than from formality in percentage points, the difference between them disappears when the percentage variation is considered.

8 Section 3.2 details the model used and the dependent and independent variables included.

9 Complete results of the regressions are available upon request.

10 This result suggests that the higher exit rates of informal workers observed in the descriptive data in this country are not be due to this condition per se, but rather to the correlation between informality and other job characteristics that have greater instability.

11 It is not intended to carry out an impact evaluation of these policies here, which is beyond the scope of this paper.

12 Based on descriptive data, Brazil experienced a decrease in exit rates from both formal and informal jobs. Therefore, in both cases, the decline in entry rates accounted for more than 100% of the decrease in employment, indicating that if only exit rates had changed, employment in Brazil would have actually increased rather than decreased. However, as shown in Fig. 2, these results change after controlling for observable variables.

13 Section 3.2 details the model used and the dependent and independent variables included.
