# Supplementary material for: Short-term labour transitions and informality during the COVID-19 pandemic in Latin America
Source: J Labour Mark Res. 2023 May 17;57(1):15. doi: 10.1186/s12651-023-00342-x (PMC10189224; doi:10.1186/s12651-023-00342-x)
Supplement: Supplementary file 1 — Additional file 1: Fig. S1. Evolution of the total employment, formal employment, informal employment and informality rate. [file 12651_2023_342_MOESM1_ESM.docx]

Figure S1. Evolution of the total employment, formal employment, informal employment and informality rate

| Argentina | Brazil |
| --- | --- |
| 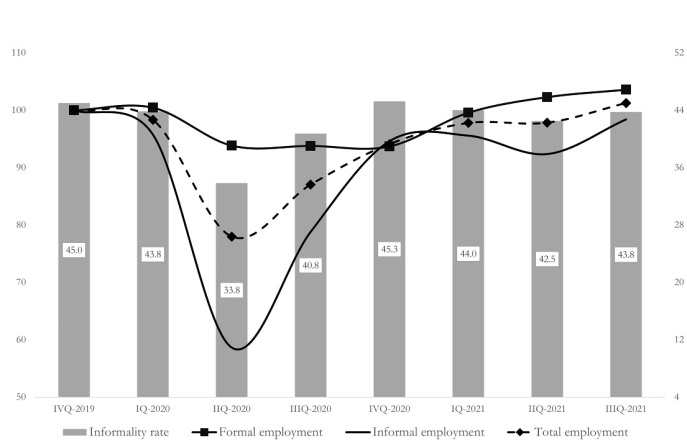 | 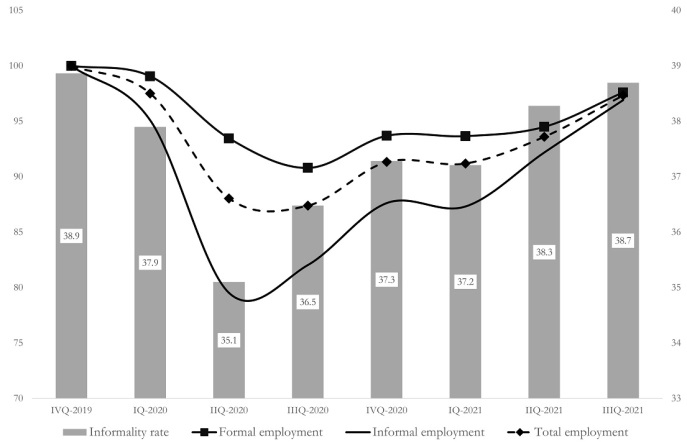 |
| Costa Rica | Mexico |
| 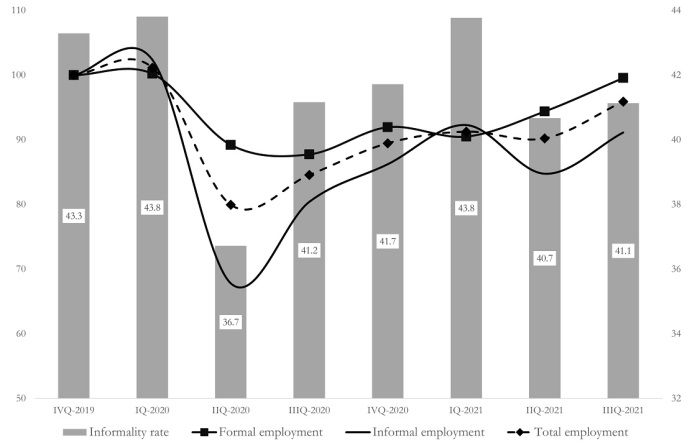 | 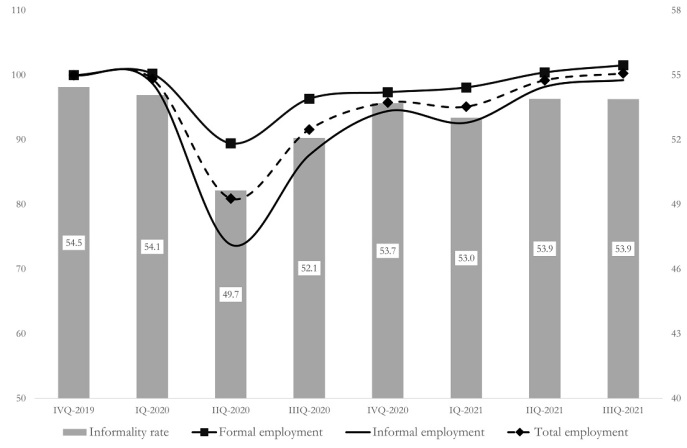 |
| Paraguay | Peru |
| 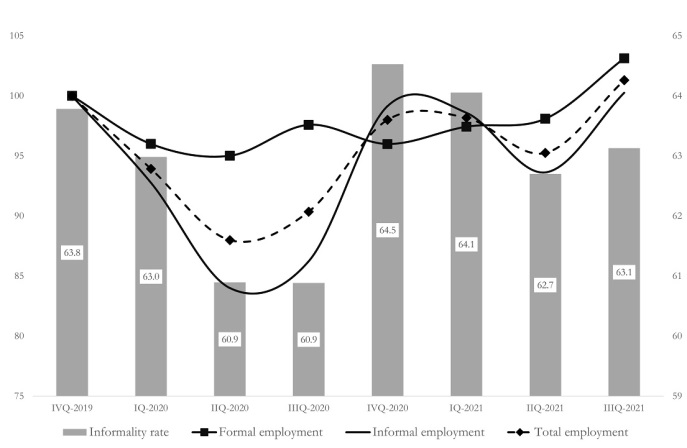 | 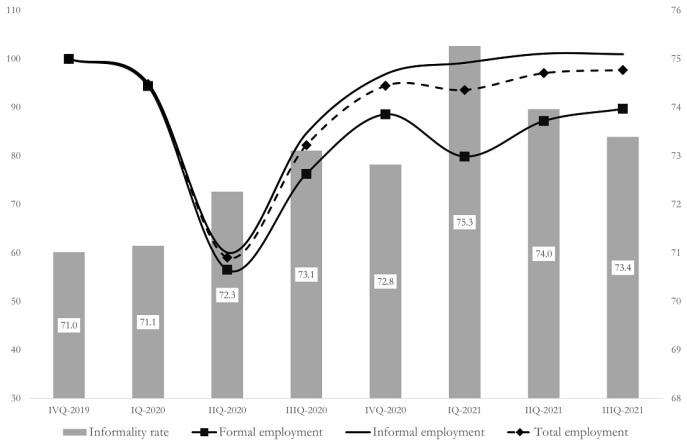 |

Source: Own elaboration based on household surveys.
