# Supplementary material for: Short-term labour transitions and informality during the COVID-19 pandemic in Latin America
Source: J Labour Mark Res. 2023 May 17;57(1):15. doi: 10.1186/s12651-023-00342-x (PMC10189224; doi:10.1186/s12651-023-00342-x)
Supplement: Supplementary file 2 — Additional file 2: Fig. S2. Contribution of formal and informal employment to total labour contraction and partial labour recovery. [file 12651_2023_342_MOESM2_ESM.docx]

Figure S2. Contribution of formal and informal employment to total labour contraction and partial labour recovery

| a) IVQ2019 – IIQ2020 | b) IIQ2020 – IIIQ2021 |
| --- | --- |
| 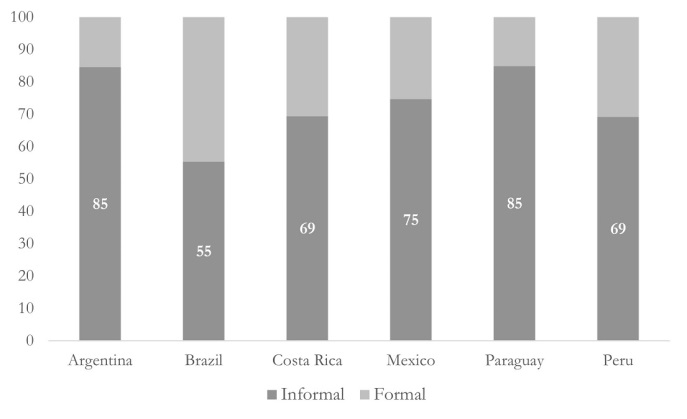 | 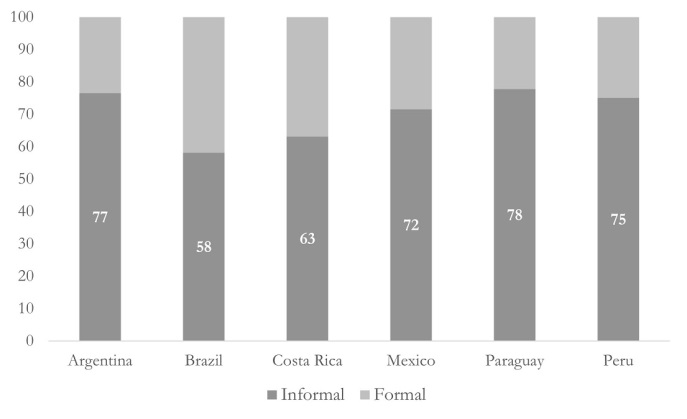 |

Source: Own elaboration based on household surveys.
