# Supplementary material for: Short-term labour transitions and informality during the COVID-19 pandemic in Latin America
Source: J Labour Mark Res. 2023 May 17;57(1):15. doi: 10.1186/s12651-023-00342-x (PMC10189224; doi:10.1186/s12651-023-00342-x)
Supplement: Supplementary file 3 — Additional file 3: Table S1. Empirical identification of informality. [file 12651_2023_342_MOESM3_ESM.docx]

Table S1. Empirical identification of informality

| Country | Salaried informal workers | Non-salaried informal workers |
| --- | --- | --- |
| Argentina | Those without health insurance (*obra social*) paid by the employer. | Identification depends on whether they have family members or associates in the firm where they work. First case: informal if the company is not duly incorporated. Second case: the place of work is taken into consideration: (1) informal own-account workers do not work in a business or office; (2) informal employers (2.1) do not work in an office, nor in a transportation vehicle, nor a construction site, nor a fixed street point; (2.2) even when working in any of these four types of establishments they have less than 5 employees. |
| Brazil | Those without a labour contract and contributions to the Institute of Social Security | Those whose firm is not registered with the National Registry of Legal Entities. |
| Costa Rica | Those whose employers do not make social security contributions | Those whose firm is not duly incorporated nor keeps formal books. |
| Mexico | Those whose employers do not make social security contributions | Those whose firm does not keep accounting records. In the case this information is missing, the place of work or size of the firm (up to 5 employees) is used. |
| Paraguay | Those who are not enrolled in the pension system. | Those whose firm does not have a taxpayer identification number. |
| Peru | Those who are not enrolled in the pension system. | Those whose firm is not registered with the National Superintendence of Customs and Tax Administration nor maintains accounting records. |

Source: Own elaboration based on ILO (2003)
