# Supplementary material for: Short-term labour transitions and informality during the COVID-19 pandemic in Latin America
Source: J Labour Mark Res. 2023 May 17;57(1):15. doi: 10.1186/s12651-023-00342-x (PMC10189224; doi:10.1186/s12651-023-00342-x)
Supplement: Supplementary file 4 — Additional file 4: Table S2. Testing for random attrition. [file 12651_2023_342_MOESM4_ESM.docx]

|  |  | I-II19 | II-III19 | III-IV19 | IV19-I20 | I-II20 | II-III20 | III-IV20 | IV20-I21 | I-II21 | II-III21 |
| --- | --- | --- | --- | --- | --- | --- | --- | --- | --- | --- | --- |
| Brazil | Pseudo R^2^ | 0.0167*** | 0.0151*** | 0.0161*** | 0.0082*** | 0.0169*** | 0.0058*** | 0.0059*** | 0.0036*** | 0.0040*** | 0.0033*** |
|  | BGLW |  | NO | NO | NO | NO | NO | NO | NO | NO | NO |
| Mexico | Pseudo R^2^ | 0.0219*** | 0.0249*** | 0.0228*** | 0.0234*** | 0.0111*** | 0.01*** | 0.01*** | 0.0064*** | 0.0081*** | 0.0091*** |
|  | BGLW | YES | YES | YES | YES | YES | YES | YES | YES | YES | YES |
|  |  |  |  |  |  |  |  |  |  |  |  |
|  |  | I19-I20 | II19-II20 | III19-III20 | IV19-IV20 | I20-I21 | II20-II21 | III20-III21 |  |  |  |
| Paraguay | Pseudo R^2^ | 0.0103*** | 0.0116*** | 0.0116*** | 0.0224*** | 0.0091*** | (*) | (*) |  |  |  |
|  | BGLW | NO | YES | YES | YES | NO | (*) | (*) |  |  |  |

Table S2. Testing for random attrition

Note: *** p<0.01, **p<0.050, * p<0.1, (*) Identification of attrition was not possible for these periods.

Source: Own elaboration based on household surveys.
