# Supplementary material for: Short-term labour transitions and informality during the COVID-19 pandemic in Latin America
Source: J Labour Mark Res. 2023 May 17;57(1):15. doi: 10.1186/s12651-023-00342-x (PMC10189224; doi:10.1186/s12651-023-00342-x)
Supplement: Supplementary file 5 — Additional file 5: Table S3. Reweighted and not-reweighted transition matrices to correct attrition bias. [file 12651_2023_342_MOESM5_ESM.docx]

Table S3. Reweighted and not-reweighted transition matrices to correct attrition bias

| (a) Mexico | | | | | | | | | |
| --- | --- | --- | --- | --- | --- | --- | --- | --- | --- |
|  | | Without adjustment | | | | With adjustment | | | |
|  |  | E | U | I | Total | E | U | I | Total |
| I19-II19 | Employed (E) | 49.8 | 1.0 | 6.5 | 57.3 | 49.9 | 1.0 | 6.6 | 57.5 |
|  | Unemployed (U) | 1.0 | 0.4 | 0.5 | 1.9 | 1.0 | 0.3 | 0.5 | 1.9 |
|  | Inactive (I) | 6.9 | 0.6 | 33.3 | 40.7 | 6.6 | 0.6 | 33.5 | 40.7 |
|  | Total | 57.7 | 2.0 | 40.3 | 100 | 57.5 | 1.9 | 40.6 | 100 |
| II19-III19 | Employed (E) | 50.1 | 1.0 | 6.8 | 58.0 | 50.0 | 1.0 | 7.0 | 58.0 |
|  | Unemployed (U) | 1.1 | 0.4 | 0.6 | 2.1 | 1.0 | 0.4 | 0.6 | 2.0 |
|  | Inactive (I) | 6.7 | 0.7 | 32.5 | 39.9 | 6.4 | 0.7 | 32.9 | 40.0 |
|  | Total | 57.9 | 2.2 | 39.9 | 100 | 57.5 | 2.1 | 40.4 | 100 |
| III19-IV19 | Employed (E) | 50.4 | 0.9 | 6.9 | 58.2 | 50.5 | 0.9 | 7.0 | 58.4 |
|  | Unemployed (U) | 1.2 | 0.4 | 0.7 | 2.3 | 1.1 | 0.4 | 0.6 | 2.1 |
|  | Inactive (I) | 6.7 | 0.6 | 32.2 | 39.5 | 6.5 | 0.6 | 32.4 | 39.5 |
|  | Total | 58.3 | 1.9 | 39.8 | 100 | 58.1 | 1.9 | 40.0 | 100 |
| IV19-I20 | Employed (E) | 50.3 | 1.0 | 7.1 | 58.4 | 50.4 | 1.0 | 7.3 | 58.7 |
|  | Unemployed (U) | 1.0 | 0.4 | 0.6 | 2.0 | 0.9 | 0.4 | 0.6 | 1.9 |
|  | Inactive (I) | 6.3 | 0.5 | 32.8 | 39.6 | 6.1 | 0.5 | 32.8 | 39.4 |
|  | Total | 57.6 | 1.9 | 40.5 | 100 | 57.4 | 1.9 | 40.7 | 100 |
| I20-II20 | Employed (E) | 46.2 | 1.4 | 11.5 | 59.1 | 46.0 | 1.4 | 11.6 | 59.0 |
|  | Unemployed (U) | 0.7 | 0.3 | 0.6 | 1.6 | 0.7 | 0.3 | 0.6 | 1.6 |
|  | Inactive (I) | 5.3 | 0.4 | 33.6 | 39.3 | 5.3 | 0.4 | 33.7 | 39.4 |
|  | Total | 52.2 | 2.1 | 45.7 | 100 | 52.0 | 2.1 | 45.9 | 100 |
| II20-III20 | Employed (E) | 43.1 | 1.1 | 8.5 | 52.6 | 43.0 | 1.1 | 8.5 | 52.6 |
|  | Unemployed (U) | 0.6 | 0.2 | 0.2 | 1.0 | 0.6 | 0.2 | 0.2 | 1.0 |
|  | Inactive (I) | 9.1 | 0.8 | 36.5 | 46.4 | 9.1 | 0.8 | 36.5 | 46.4 |
|  | Total | 52.8 | 2.1 | 45.2 | 100 | 52.7 | 2.1 | 45.2 | 100 |
| III20-IV20 | Employed (E) | 45.1 | 0.9 | 6.7 | 52.7 | 45.2 | 0.9 | 6.7 | 52.7 |
|  | Unemployed (U) | 1.4 | 0.5 | 0.8 | 2.7 | 1.4 | 0.5 | 0.8 | 2.7 |
|  | Inactive (I) | 8.3 | 1.0 | 35.3 | 44.6 | 8.3 | 1.0 | 35.3 | 44.6 |
|  | Total | 54.8 | 2.4 | 42.8 | 100 | 54.8 | 2.3 | 42.8 | 100.0 |
| IV20-I21 | Employed (E) | 46.6 | 1.1 | 7.2 | 54.9 | 46.7 | 1.1 | 7.3 | 55.1 |
|  | Unemployed (U) | 1.1 | 0.5 | 0.9 | 2.5 | 1.1 | 0.5 | 0.8 | 2.4 |
|  | Inactive (I) | 6.8 | 0.8 | 35.0 | 42.6 | 6.8 | 0.8 | 35.0 | 42.5 |
|  | Total | 54.5 | 2.4 | 43.1 | 100 | 54.6 | 2.4 | 43.1 | 100.0 |
| I21-II21 | Employed (E) | 47.8 | 1.0 | 6.4 | 55.2 | 47.8 | 1.0 | 6.4 | 55.2 |
|  | Unemployed (U) | 1.2 | 0.4 | 0.7 | 2.3 | 1.2 | 0.4 | 0.7 | 2.3 |
|  | Inactive (I) | 7.5 | 0.8 | 34.2 | 42.5 | 7.5 | 0.8 | 34.2 | 42.5 |
|  | Total | 56.5 | 2.3 | 41.2 | 100 | 56.5 | 2.2 | 41.3 | 100 |
| II21-III21 | Employed (E) | 48.7 | 1.2 | 7.0 | 56.9 | 48.8 | 1.1 | 7.1 | 57.0 |
|  | Unemployed (U) | 1.2 | 0.4 | 0.7 | 2.3 | 1.2 | 0.4 | 0.7 | 2.3 |
|  | Inactive (I) | 7.0 | 0.7 | 33.1 | 40.8 | 6.9 | 0.7 | 33.1 | 40.7 |
|  | Total | 56.9 | 2.3 | 40.8 | 100 | 56.9 | 2.2 | 40.9 | 100 |
| (b) Paraguay | | | | | | | | | |
|  | | Without adjustment | | | | With adjustment | | | |
|  |  | E | U | I | Total | E | U | I | Total |
| I19-I20 | Employed (E) | 57.8 | 2.5 | 7.4 | 67.7 | 57.9 | 2.5 | 7.5 | 67.9 |
|  | Unemployed (U) | 2.5 | 1.0 | 1.4 | 4.9 | 2.5 | 1.0 | 1.4 | 4.9 |
|  | Inactive (I) | 6.4 | 1.6 | 19.4 | 27.4 | 6.3 | 1.6 | 19.3 | 27.2 |
|  | Total | 66.7 | 5.1 | 28.2 | 100 | 66.7 | 5.1 | 28.2 | 100 |
| II19-II20 | Employed (E) | 56.2 | 3.2 | 9.9 | 69.3 | 56.4 | 3.2 | 9.8 | 69.4 |
|  | Unemployed (U) | 1.7 | 0.8 | 1.5 | 4.0 | 1.7 | 0.7 | 1.5 | 3.9 |
|  | Inactive (I) | 4.8 | 1.1 | 20.9 | 26.7 | 4.8 | 1.1 | 20.8 | 26.7 |
|  | Total | 62.7 | 5.1 | 32.2 | 100 | 62.9 | 5.0 | 32.1 | 100 |
| III19-III20 | Employed (E) | 57.3 | 3.2 | 7.2 | 67.7 | 57.5 | 3.1 | 7.1 | 67.7 |
|  | Unemployed (U) | 2.1 | 0.8 | 1.4 | 4.3 | 2.1 | 0.8 | 1.4 | 4.3 |
|  | Inactive (I) | 7.2 | 1.4 | 19.4 | 28.0 | 7.3 | 1.4 | 19.3 | 28.0 |
|  | Total | 66.6 | 5.4 | 28.0 | 100 | 66.9 | 5.3 | 27.8 | 100 |
| IV19-IV20 | Employed (E) | 58.9 | 2.4 | 6.9 | 68.2 | 59.4 | 2.3 | 7.1 | 68.8 |
|  | Unemployed (U) | 2.0 | 0.8 | 0.9 | 3.7 | 2.0 | 0.7 | 1.0 | 3.7 |
|  | Inactive (I) | 6.2 | 1.7 | 20.2 | 28.1 | 6.0 | 1.7 | 19.8 | 27.5 |
|  | Total | 67.1 | 4.9 | 28.0 | 100 | 67.4 | 4.7 | 27.9 | 100 |
| I20-I21 | Employed (E) | 57.6 | 2.5 | 6.2 | 66.3 | 57.9 | 2.5 | 6.3 | 66.7 |
|  | Unemployed (U) | 2.7 | 1.2 | 1.7 | 5.6 | 2.7 | 1.2 | 1.7 | 5.6 |
|  | Inactive (I) | 7.6 | 1.6 | 18.9 | 28.1 | 7.6 | 1.6 | 18.6 | 27.8 |
|  | Total | 67.9 | 5.3 | 26.8 | 100 | 68.2 | 5.3 | 26.6 | 100 |

Source: Own elaboration based on household surveys.
